# Axillo-subclavian dissection and pseudoaneurysm following inferior glenohumeral dislocation: Case report and literature review

**DOI:** 10.1016/j.ijscr.2019.11.058

**Published:** 2019-12-06

**Authors:** Adel Elkbuli, John Ehrhardt, Mark McKenney, Dessy Boneva, Stacey Martindale

**Affiliations:** a Department of Surgery, Kendall Regional Medical Center, Miami, FL, USA; b University of South Florida, Tampa, FL, USA; c Department of Surgery, Aventura Hospital and Medical Center, Miami, FL, USA

**Keywords:** CT, computed tomography, GCS, Glasgow coma score, ICU, intensive care unit, pRBCs, packed red blood cells, FFP, fresh frozen plasma, POD, post-operative day, Axillary artery dissection, Axillary pseudoaneurysm, Glenohumeral dislocation, Luxatio erecta, Ground-level fall

## Abstract

**Introduction:**

Axillo-subclavian arterial injuries are generally associated with penetrating trauma. On rare occasion, blunt mechanisms can cause these injuries in the setting of high-energy trauma, humeral head or neck fractures, and severe glenohumeral dislocations. Glenohumeral dislocations sustained from ground-level falls are generally reduced in the emergency department without associated morbidity.

**Presentation of case:**

An 80-year-old woman presented with an inferior glenohumeral dislocation after a ground-level fall that was found to be complicated by axillary dissection, pseudoaneurysm, and acute hemorrhage. Endovascular intervention with a balloon-inflatable stent successfully controlled extravasation and restored perfusion to the affected upper extremity. After a short hospitalization, the patient was discharged with intact neurovascular status.

**Discussion:**

Blunt axillary artery injury and inferior glenohumeral dislocations are both uncommon entities. A correlation exists between inferior dislocations and neurovascular complications. Accordingly, there may be a role for diagnostic vascular imaging for patients with inferior glenohumeral dislocations. Endovascular stenting was successful in our case and backed by case series and cohort studies in the literature. Some controversy exists regarding stent patency and follow-up planning in trauma patients.

**Conclusion:**

This case represents a critical vascular injury from an unexpected mechanism. Inferior glenohumeral dislocations, regardless of injury mechanism, should raise the index of suspicion for vascular involvement. Endovascular repair in our patient was life-saving given her advanced age, acute blood loss anemia, rarity and severity of her injuries and multiple medical comorbidities.

## Highlights

• This is a rare case of Axillo-subclavian dissection and pseudoaneurysm following blunt chest injury.
• Inferior shoulder dislocation is uncommon but may cause serious vascular injury and life-threatening hemorrhage.
• An endovascular approach was life-saving for our patient considering her advanced age and medical comorbidities.

## 1 Introduction

Glenohumeral dislocations are common with blunt shoulder trauma. The vast majority of injuries undergo closed reduction in the emergency department without complication or operative intervention. Patients almost invariably recover with conservative management after a period of upper extremity sling immobilization and targeted physical therapy. Arterial injury is rare in the absence of fractures with low-energy musculoskeletal trauma, occurring in only 1–2 % of glenohumeral dislocations [1].

**Fig. 1:**
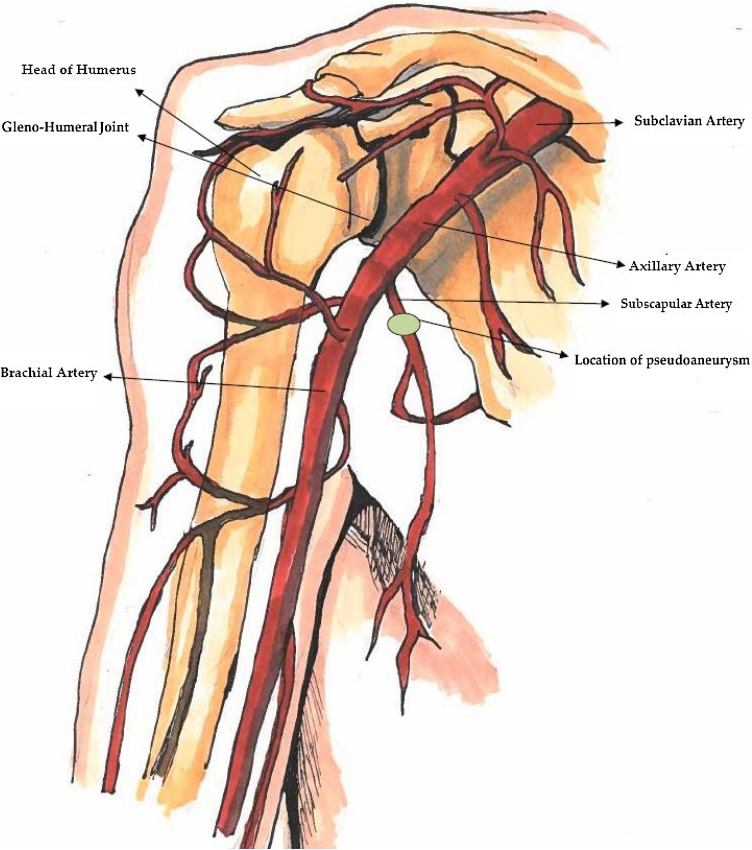
Anatomy of the Shoulder: Subclavian artery (arrow), axillary artery (arrow), subscapular artery (arrow), location of pseudoaneurysm (circle and arrow).

Inferior glenohumeral dislocations (*luxatio erecta*) are a rare subtype representing less than 0.5 % of all shoulder dislocations [2]. These injuries are believed to carry the highest probability of vascular compromise [3,4]. Regional anatomic relationships account for the mechanism of axillo-subclavian involvement with these dislocations. The axillary artery and brachial plexus enter the upper extremity inferior to the glenohumeral joint (Fig. 1).

Ground-level falls are a significant cause of morbidity, mortality, and healthcare expenditures with an aging population [5]. Falls are a common mechanism of injury and carry an associated mortality that is higher than many other injuries [6]. We present the case of an 80-year-old woman who suffered a traumatic inferior glenohumeral dislocation during a ground-level fall. Following routine reduction in the emergency department, the patient decompensated over the coming hours and ultimately required an emergency endovascular operation for active extravasation from the axillary artery. This case is reported with consideration to the SCARE criteria [7].

## 2 Presentation of case

**Fig. 2:**
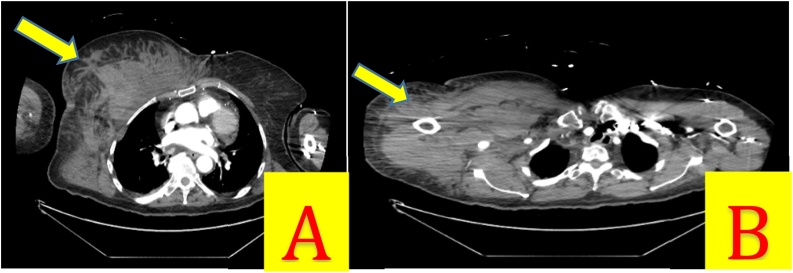
A) Large right chest wall hematoma. B) Large right shoulder hematoma.

**Fig. 3:**
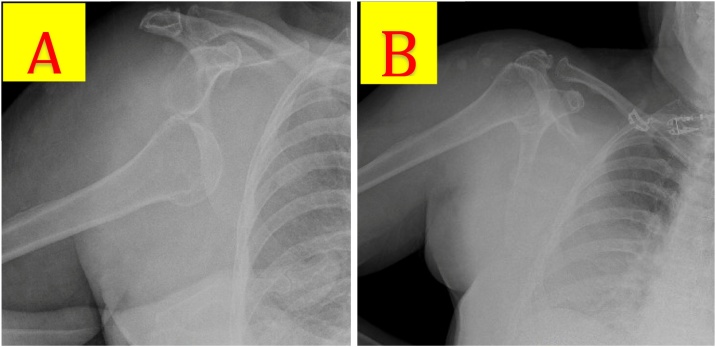
A) Index right shoulder radiograph: Inferior glenohumeral dislocation with greater humeral tuberosity abutting the infraglenoid tubercle. B) Post-reduction radiograph with no acute fractures or deformities.

An 80-year-old woman with atrial fibrillation anticoagulated on apixaban presented to the emergency department complaining of right shoulder pain after an unwitnessed ground-level fall. She could not recall the fall but her daughter endorsed that it happened in the middle of the night and she found her mother lying on her floor, screaming on her right side. The patient’s home medications included levothyroxine, losartan, apixaban, glipizide, memantine, atenolol, rosuvastatin, and linagliptin. Her vital signs on arrival were in the normal range, and her oxygen saturation was 100 % on room air. Her injuries included a right chest wall and shoulder hematoma (Fig. 2), bilateral periorbital hematomas, concussion with short loss of consciousness, and an acute, first-time right glenohumeral joint dislocation. CT head showed no intracranial hemorrhage and CT neck was without acute injury. Right shoulder radiograph demonstrated an inferior dislocation of a degenerative glenohumeral joint without fracture (Fig. 3A). Her upper extremity neurovascular exam was normal and her shoulder was reduced in the emergency department without difficulty. Post-reduction radiograph showed the humeral head in the glenoid cavity (Fig. 3B); the patient had intact neurovascular status. She was admitted for observation based on her advanced age and comorbid conditions including dementia, hypertension, hyperlipidemia, hypothyroidism, atrial fibrillation on therapeutic anticoagulation, functional dependence, and obesity.

**Fig. 4:**
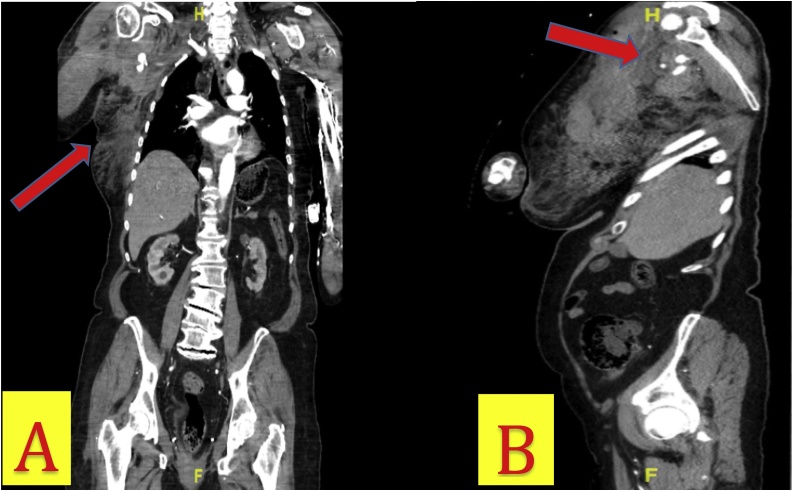
A) CT Angiography of the chest and abdomen (coronal view) demonstrating right subclavian artery dissection, 2.4-cm axillary artery bilobed pseudoaneurysm, possible proximal brachial artery injury, and large right chest wall hematoma. B) CT Angiography of the chest and abdomen (sagittal view) showing large right shoulder hematoma and pseudoaneurysm in proximity to the humeral head of the right post-reduction. Pseudoaneurysm is positioned in the inferior location to the gleno-humoral joint.

**Fig. 5:**
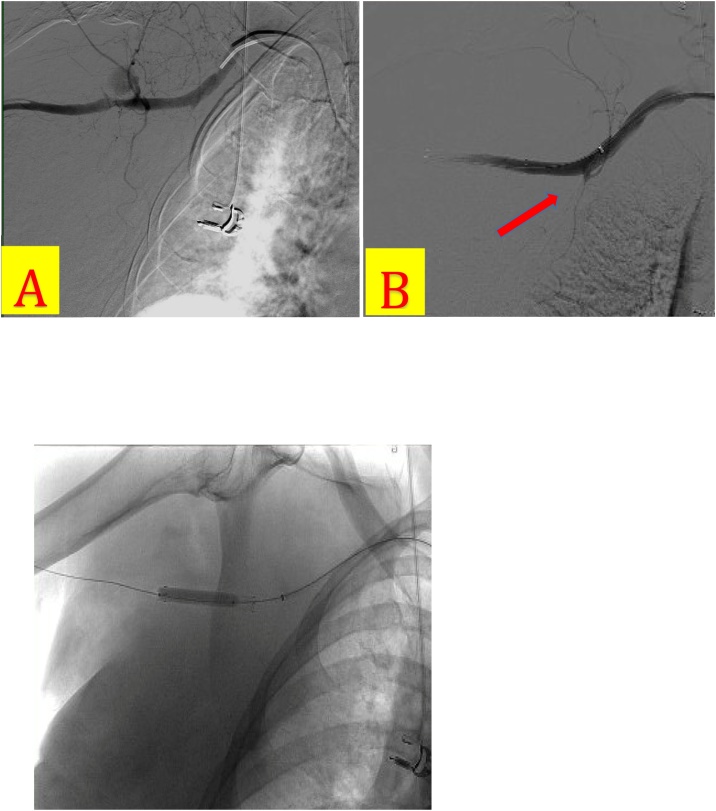
A) Intraoperative axillo-subclavian arteriogram with pseudoaneurysm blush at the level of the distal axillary artery. B) Stent placement arteriogram showing endovascular prosthesis effectively covering pseudoaneurysm.

**Fig. 6:**
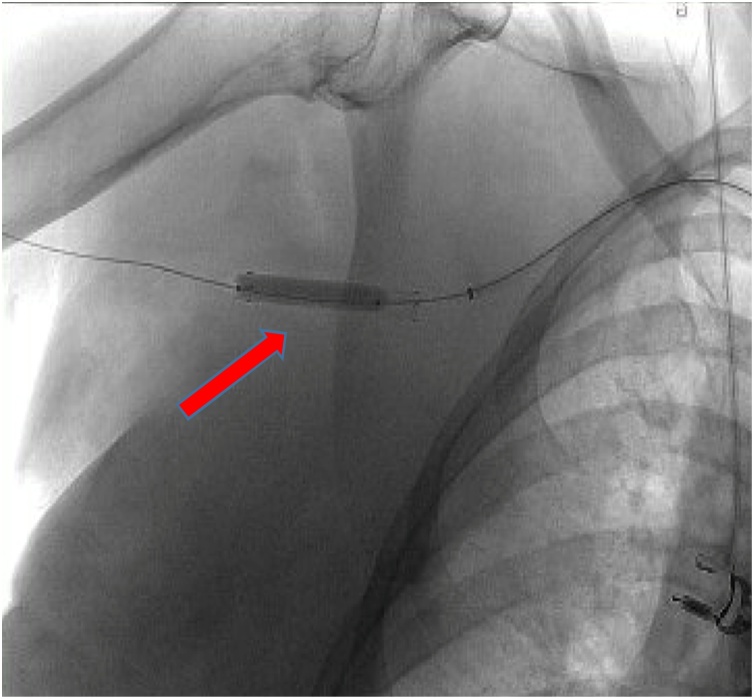
Intraoperative radiograph post-endovascular deployment of balloon-inflatable prosthesis over guide wire in distal axillary artery.

Three hours later, the patient became hemodynamically unstable, with a blood pressure of 73/46 mm Hg and an enlarging chest wall hematoma. She did not have a palpable right radial pulse. Resuscitation with an isotonic crystalloid bolus restored the patient’s blood pressure to 125/65. CT angiography demonstrated right subclavian artery dissection, 2.4-cm axillary artery bilobed pseudoaneurysm with active extravasation, and the large right chest wall hematoma (Fig. 4A&B). Follow up hemoglobin level dropped from 11.6 to 6.5 g/dL. She was taken to the operating room for emergent control of the active hemorrhage. Intra-operative subclavian and axillary angiogram demonstrated the active extravasation of subclavian and axillary branches (Fig. 5A&B). A balloon-expandable endoprosthesis (Gore® Viabahn®) with heparin bioactive surface was placed via percutaneous arterial access (Fig. 6). Post-stent angiogram ensured that distal flow to radial and ulnar arteries was preserved. The patient tolerated the procedure well and received two units of blood.

Post operatively she did well and her oral apixaban was restarted on POD 1 to prevent stent thrombosis. On POD 5, she was discharged to an inpatient rehabilitation facility. Follow up at 2 weeks in the clinic for clinical and duplex stent surveillance demonstrated no neurovascular compromise.

## 3 Discussion

Axillary arterial injury following glenohumeral dislocation was first reported in 1911 by French surgeon Maurice Guibé. His manuscript *Des lesions des vaisseaux de l’aisselle qui compliquant les luxation de l’epaule* (Lesions of axillary vessels which complicate shoulder dislocations) compiled fifty-seven cases [8]. Arterial ligation was the standard care at that time, now largely superseded by endovascular intervention.

Current literature shows that over 90 % of axillo-subclavian injuries occur from penetrating trauma [9]. Blunt trauma represents only a minor subset of these injuries and tend to be associated with high-energy impact (e.g., motor vehicle collisions, falls from significant height) [10] and fracture or severe dislocation [11]. In general, the injury pattern is a reflection of the force and mechanism of injury. The classic case for an axillo-subclavian artery injury requiring emergent intervention is (1) a patient with recurrent or high-force anterior dislocation accompanied by (2) expanding axillary hematoma and (3) diminishing pulses in the affected upper extremity. This pathognomonic triad often declares itself early during initial evaluation. Our case represents a rare vascular injury, especially in the context of a low-energy blunt mechanism—a ground-level fall in our patient—that developed over several hours before becoming apparent.

Certain demographics should raise the index of suspicion for vascular involvement. Patients with multiple prior dislocations should be evaluated more judiciously for these rare injuries. Sports-related glenohumeral dislocations (ice hockey, thoroughbred horse riding, baseball, volleyball, and cycling) place patients at higher risk for axillo-subclavian complications [[12], [13], [14]]. Shoulder dislocations in patients over the age of fifty also have higher likelihood of vascular compromise. Gender does not seem to predispose to glenohumeral instability. Our patient was eighty years old, but she had never had a shoulder dislocation in the past and the mechanism of injury was low energy compared to others with associated vascular injury [15].

Directionality of dislocation also plays an important role in neurovascular involvement. Anterior dislocations are the most common (95–97 %) type of dislocation but are generally uncomplicated. Reports of arterial injury with anterior dislocations are mainly due to the fact that they constitute the vast majority of cases. Posterior dislocations are far less common (2–5 %) and only rarely cause neurovascular disruption [16]. Inferior dislocations are the least common (<0.5 %) type of dislocation, often caused by hyperabduction, and pose the highest risk for axillary artery and brachial plexus injury [17]. Our patient’s dislocation was inferior and complicated by axillary artery involvement.

Overall, less than 1 % of glenohumeral dislocations presenting to trauma centers involve arterial injury [18]. Iakovlev et al. presented an axillary pseudoaneurysm in 2014 with a similar mechanism (ground-level fall) and pattern of injury to our patient [19]. Their patient had five previous anterior shoulder dislocations before suffering an inferior dislocation that caused axillary arterial hemorrhage. Conversely, our patient sustained this rare injury along with her first-ever dislocation. In terms of operative management, both cases accomplished endovascular repair with a balloon-inflatable stent.

Although heterogeneity exists with endovascular repair, some institutions have developed standardized methods based on their experiences. Barão et al. discussed the details of their endovascular approach for traumatic subclavian injuries in fifteen cases. They obtained femoral artery access for the procedure. Intraoperative intravenous heparin was not used because of the perceived risk of bleeding from concurrent axillary venous injuries. The stent ideally covered 1.5–2-cm of healthy artery proximal and distal to the injury. For complete axillo-subclavian transections complicated by hematoma, they performed a brachial artery cut-down and snared the lodged femoral catheter at the level of the transection [20].

Outcomes of endovascular stenting versus open repair for traumatic axillo-subclavian injuries have been addressed by retrospective cohort studies. Branco et al. evaluated 18 endovascular interventions alongside 135 demographically-matched open repairs and found improved mortality and lower complication rates for endovascular intervention patients [21]. Ganapathy et al. studied 20 endovascular interventions alongside 50 open repairs for subclavian, axillary, femoral, and popliteal injuries. They found that endovascular repairs required subsequent fasciotomy less often, had lower transfusion requirements, higher limb salvage rates, yet led to longer hospitalizations (9 vs. 14 days) [22].

One valid argument against endovascular stenting is the possibility of stent occlusion. Long-term antiplatelet therapy is the standard of care following endovascular stenting. Our patient had excellent adherence to a novel oral anticoagulant, so we were less concerned about stent thrombosis. Nonetheless, surgeons at urban trauma centers understand that patients may not routinely followed-up in clinic or adhere to their antiplatelet therapy regimen. A retrospective review of the National Trauma Data Bank (NTDB) demonstrated that axillo-subclavian covered stent occlusion occurred in 30 % of patients at a median time of 132 days. For this reason, open repair requiring only one month of dual-antiplatelet therapy—P2Y_12_-ADP receptor antagonist (e.g. clopidogrel, ticagrelor) plus aspirin—will always have a role and may be more suitable for some patients [23].

## 4 Conclusion

We presented an uncommon case of inferior glenohumeral dislocation complicated by axillo-subclavian dissection and pseudoaneurysm. Elderly patients with ground-level fall and active hemorrhage carry a high mortality risk. An endovascular approach was life-saving for our patient considering her advanced age and medical comorbidities. Inferior dislocations are a rare subset with higher risk of neurovascular injury. For this reason, patients with these dislocations should be thoroughly and serially assessed for focal neural deficits and vascular status changes. When these patients are hemodynamically stable, CT angiography is merited for definitive evaluation of the axillary vessels. Endovascular intervention and repair with balloon-inflatable stents can be safe and effective, even in the presence of hemodynamic instability. Although endovascular approaches are becoming more common, open repair remain effective and relevant, especially for younger trauma patients with uncertain follow-up planning.

## Sources of funding

None.

## Ethical approval

This is a case report study. This report was conducted in compliance with institutional ethical standards. Informed written consent and ethical approval has been obtained and all identifying information was omitted.

## Consent

Informed written consent has been obtained and all identifying information is omitted.

## Author contributions

AE, DB, SM – Conception of study, acquisition of data, analysis and interpretation of data

SM, DB – Management of case AE, JE, DB, MM, SM –drafting of abstract, drafting of manuscript, critical revision of manuscript.

AE, JE, DB, MM. SM – Approval of the final version for submission

## Registration of research studies

This is a case report study.

## Guarantor

Dessy Boneva.

Stacey Martindale.

## Provenance and peer review

Not commissioned, externally peer-reviewed.

## Declaration of Competing Interest

None.
